# Brazilian conditional cash transfer programme’s impact on youth human capital outcomes: the 2004 Pelotas Birth Cohort

**DOI:** 10.1136/bmjph-2025-003192

**Published:** 2025-11-10

**Authors:** Jessica Mayumi Maruyama, Cristiane Silvestre Paula, Carolina Ziebold, Luciana Tovo-Rodrigues, Iná S Santos, Aluísio J D Barros, Joseph Murray, Sara Evans-Lacko, Alicia Matijasevich

**Affiliations:** 1 Graduate Program in Human Developmental Sciences, Mackenzie Presbyterian University, São Paulo, Brazil; 2 Departamento de Psiquiatria, Universidade Federal de São Paulo, Sao Paulo, Brazil; 3 Postgraduate Program in Epidemiology, Federal University of Pelotas, Pelotas, Brazil; 4 Human and Violence Research Centre (DOVE), Federal University of Pelotas, Pelotas, Brazil; 5 Care Policy and Evaluation Centre, The London School of Economics and Political Science, London, UK; 6 Departamento de Medicina Preventiva, Universidade de São Paulo, São Paulo, Brazil

**Keywords:** Adolescent, Public Health, Preventive Medicine

## Abstract

**Introduction:**

Brazil’s Bolsa Família Programme (BFP) is one of the largest conditional cash transfer (CCT) programmes globally. This study evaluated the impact of BFP during childhood on human capital outcomes at age 18.

**Methods:**

We analysed data from 2743 participants in the 2004 Pelotas Birth Cohort. BFP participation was assessed at ages 6 and 11, and outcomes at age 18 included grade repetition, being neither in school nor working, criminal behaviour, tobacco use, binge drinking and drug use. To address selection bias, we used Propensity Score Matching to estimate the average treatment effect on the treated (ATT).

**Results:**

No significant effects of BFP participation at ages 6 or 11 were observed for most outcomes: not studying or working (ATT 0.01; 95% CI −0.04 to 0.06), tobacco use (ATT −0.03; 95% CI −0.10 to 0.05), binge drinking (ATT −0.05; 95% CI −0.15 to 0.05), violent crime (ATT 0.00; 95% CI −0.08 to 0.07) or any crime (ATT −0.06; 95% CI −0.14 to 0.03). Weak evidence suggested reductions in non-violent crime (ATT −0.04; 95% CI −0.09 to 0.01; p=0.072) and drug use (ATT −0.07; 95% CI −0.17 to 0.02; p=0.075), but a possible increase in grade repetition (ATT 0.07; 95% CI −0.02 to 0.16; p=0.097). Analyses of BFP receipt at only one time point showed no effects, indicating that longer exposure may be necessary for impact.

**Conclusions:**

Participation in the BFP during childhood was not associated with significant improvements in most human capital outcomes at age 18. Nonetheless, potential reductions in drug use and non-violent crime, and the complex relationship with school performance, warrant further investigation into the long-term and multidimensional effects of CCTs. These results are specific to the Pelotas cohort and may not be generalisable to different geographical settings within Brazil.

**WHAT IS ALREADY KNOWN ON THIS TOPIC**

- Brazil’s conditional cash transfer programme, Bolsa Família, has demonstrated positive effects on poverty reduction, health and educational indicators. However, its long-term impact on broader youth human capital outcomes—such as school engagement, risk behaviours and crime—remains unclear.

**WHAT THIS STUDY ADDS**

- This study found no strong evidence that receiving Bolsa Família in childhood significantly affected most youth human capital outcomes at age 18. Nonetheless, there was weak evidence suggesting reduced risk for non-violent crime and drug use, and a possible increase in grade repetition among beneficiaries.

**HOW THIS STUDY MIGHT AFFECT RESEARCH, PRACTICE OR POLICY**

- The findings underscore the need to assess conditional cash transfer programmes beyond their short-term goals. For greater impact on adolescent development, policies may require complementary strategies that support school retention and reduce risk behaviours during the transition to adulthood.

## Introduction

 Conditional Cash Transfer (CCT) programmes are widely used social welfare initiatives aimed at mitigating poverty and reducing disparities by providing targeted financial aid to low-income families on meeting specific conditions.^1 2^ These programmes have gained global traction as an approach to combat poverty and inequality while promoting positive behaviours like education attainment and healthcare utilisation. CCT programmes may address the immediate needs of low-income households while also targeting root causes of poverty and inequality.^1 2^ At their core, these programmes focus on breaking the intergenerational cycle of poverty by directing resources towards investing in human capital, often with a particular emphasis on incentivising parents to ensure their children consistently attend school and undergo routine health check-ups.^1^,^3^

Initiated in 2003, Brazil’s Bolsa Família Programme (BFP) is one of the most widely recognised and largest CCT programmes worldwide, in terms of coverage and financing.^4 5^ The programme encompassed more than 14 million households, roughly equivalent to 20% of the Brazilian population, with government investment amounting to 0.4% of the gross domestic product (GDP). Eligibility for the programme is determined based on per capita household income and household composition, prioritising pregnant and breastfeeding mothers and families with children and adolescents aged 0–17. The conditions associated with the benefit include attending prenatal care visits, adhering to the national vaccination schedule, monitoring the nutritional status of children under 7 years old and ensuring school enrolment and attendance. For children aged 4–5, a minimum school attendance rate of 60% is required, while beneficiaries aged 6–17 who have not completed basic education must maintain at least 75% attendance.^5 6^ In response to the COVID-19 pandemic, the federal government introduced the Auxílio Emergencial, a distinct emergency cash transfer programme created in 2020 to provide temporary financial support to informal workers, unemployed individuals and low-income populations not necessarily covered by Bolsa Família. At the same time, Bolsa Família benefits were expanded both vertically (ie, increasing payments to existing beneficiaries) and horizontally (eg, expanding coverage).^6 7^ In 2023, the BFP was reformulated by the federal government. The revised BFP structure involves the disbursement of R$142 (approximately US$25.8, considering the exchange rate of R$5.50 per US dollar) per individual along with a supplementary benefit to ensure a minimum family income of R$600 (US$109). Furthermore, a payment of R$150 (US$27.3) is made for each child aged 0–6 years, called the early childhood benefit. An additional family variable benefit is paid to families including pregnant women and/or children aged 7–12 years and/or adolescents aged 12–18 years old; in the value of R$50 per person meeting these criteria.^8^

Since its implementation, several studies have examined the effects of the BFP on health and educational outcomes of beneficiaries and their families.^3 4 9^ These studies found that the BFP has had a positive impact on child vaccination coverage, reduced maternal and child mortality, reduced preterm births and reduced incidence and death from diarrhoea and malnutrition.^9^,^14^ Additional benefits have been reported in relation to reduced mortality from oral cancer, lower incidence of malaria and decreased hospitalisations due to HIV/AIDS.^15^,^17^ A recent scoping review further summarised the available evidence on these findings, highlighting consistent evidence for the programme’s positive effects on poverty reduction, employment, school dropout and attendance, and mortality among children and adults, as well as on violence-related outcomes such as homicide, suicide, crime and hospitalisations.^9^ However, the same review also pointed to mixed or null effects for certain outcomes, such as no impact on teenage pregnancy and some evidence of increased intimate partner violence and reinforcement of gender stereotypes among women. Some studies have specifically examined the effects of the BFP on child and adolescent mental health, with mixed results—while some found a protective association,^15 18^ others found no significant impact.^19^ Rasella *et al* discussed the possible mechanisms by which receipt of CCT policies such as the BFP cause positive effects on a wide range of aspects of child survival and development, both through direct and indirect effects. These mechanisms include improving nutrition, hygiene practices and routine check-ups and care-seeking for illness.^12^ More recently, Evans-Lacko *et al* presented a conceptual framework highlighting various mechanisms through which cash transfer programmes can enhance the mental health and prospects of young people. They propose that financial stability from such programmes can alleviate economic stress, subsequently reducing household tensions and conflicts, which benefits the mental health of all family members. Moreover, the financial support provided by CCTs can boost self-esteem and social participation, as beneficiaries are able to purchase better clothing and other necessities, thus diminishing feelings of shame and fostering self-worth. This improvement in self-esteem can lead to increased social interaction and stronger social relationships, positively affecting mental health. On a broader scale, CCTs can mitigate community violence and social exclusion by fostering social trust and strengthening communal bonds.^20^

Most studies assessing the impact of the BFP have focused on childhood or early adolescence, while emerging evidence suggests that measuring long-term outcomes of CTPs is essential for understanding the sustainability of the achieved results.^21^ Considering that the implementation of the BFP has reached its 20th anniversary, potential beneficial effects in breaking the intergenerational cycle of poverty should now be tested, as the first generation of children participating in the programme has reached early adulthood and productive age. For instance, the programme appears to have a positive impact on employability, primarily by contributing to an increase in the number of individuals participating in the labour market.^9^ Investigating potential gains in human capital into adulthood, considering outcomes such as school success and involvement in crime, is essential for public policy governance.^22^,^26^ Another important point to consider when analysing the success of cash transfer programmes is their effect in avoiding the adoption of risky behaviours that can have longer-term, life-course implications, especially if initiated in adolescence or early adulthood. Various risky behaviours, such as alcohol and substance use, are also transmitted across generations, creating a cascade effect of vulnerability accumulation beyond an individual’s own long-term health outcomes.^26 27^ In this study, we adopt a broad perspective of human capital, acknowledging that it is a multidimensional construct discussed across different theoretical traditions.^2628^,^34^ While classical economic approaches emphasise education, skills and productivity,^28 29^ more recent perspectives incorporate health, behaviours and social participation as key components.^30^,^33^ From this standpoint, outcomes such as grade repetition and being neither in school nor working are considered indicators of educational accumulation and engagement in productive activities, whereas criminal involvement and health-risk behaviours (tobacco use, binge drinking, drug use) are viewed as potential constraints on human capital development across the life course.^2631^,^34^ This broader framework allows us to situate our analysis within the interdisciplinary literature on human capital, while recognising that alternative conceptualisations may highlight different dimensions of these same outcomes. Therefore, in addition to the key outcomes primarily targeted in poverty reduction, it is important to investigate whether cash transfer programmes also affect a broader view of the concept of human capital by focusing on social and health risk behaviours of individuals.

The present study aimed to evaluate the impact of receiving the BFP during childhood on human capital outcomes at 18 years old, using data from the 2004 Pelotas Birth Cohort. Specifically, we examined the effects of receiving the benefit at ages 6 and 11 separately, as well as at both time points jointly. The outcomes assessed included grade repetition, being neither in education nor employment, involvement in non-violent and violent crime, tobacco use, binge drinking and drug use.

## Materials and methods

### Study site

The 2004 Pelotas Birth Cohort is a prospective, longitudinal study encompassing all live births from 1 January 2004 to 31 December 2004, in Pelotas, Brazil. Pelotas is a medium-sized city in the southern state of Rio Grande do Sul, Brazil, with an estimated population of approximately 336 150 inhabitants in 2025.^35^ In Pelotas, commerce, services and industry constitute the main economic sectors.^35^ Although in 1982 the city’s GDP exceeded the national average by 9%, by 2004 it had fallen to 39% below the Brazilian mean, illustrating a relative economic downturn.^36^ Regarding social indicators, school attendance among children aged 6–14 reached 98.76% in 2022, placing Pelotas 3477th out of 5570 in Brazil.^35^ Additionally, Pelotas exhibited a Human Development Index of 0.739 compared with the national average of 0.727, marginally higher primary school completion (58.0% vs 54.9%), but lower per capita GDP (R$21 553 vs R$29 466) and higher homicide rates (33.5 vs 28.4 per 100 000).^37^ Data from the 2004 Pelotas Birth Cohort further show that BFP coverage expanded considerably between 2004 and 2010, from 29% to 63%, while targeting efficiency (measured as the proportion of eligible families among beneficiaries) remained stable at around 37%.^38^ In August 2025, administrative records indicated that 21 891 families in Pelotas received Bolsa Família benefits, corresponding to 52 472 individuals, with a total investment of R$14 370 947.00 (approximately US$2 613 809) and an average benefit of R$656.48 per family (about US$119.36)^39^

### Participants

In 2004, a total of 4231 newborns, equivalent to 99.2% of the year’s total births from mothers residing in the urban area, were included in the study. During the perinatal study, trained interviewers administered standard and precodified questionnaires to mothers in the hospital after delivery. These questionnaires covered socioeconomic, demographic, behavioural and other relevant characteristics. Subsequent assessments of cohort participants occurred at ages 3, 12, 24 and 48 months, as well as at ages 6 and 11 years, with follow-up rates of 95.7%, 94.3%, 93.5%, 92.0%, 90.2% and 86.6%, respectively. The 18-year follow-up, conducted between February and December 2022, achieved a follow-up rate of 85.0%. Comprehensive details about the cohort’s methodology and follow-up interviews can be found in previous publications.^40^,^42^

For the present analysis, we excluded participants who had missing information on the socioeconomic and demographics variables assessed at the perinatal and 48-month follow-ups and those belonging to the wealthiest strata, as they were in principle not eligible for the programme and a very small proportion of them reported being a BFP beneficiary (1.25%).^19^ In addition, we also excluded participants lost to follow-up in the 18-year wave. Online supplemental figure S1 presents the flow chart of included/excluded participants.

### Patient and public involvement

Participants and members of the public were not involved in the design, conduct, reporting or dissemination plans of this research. The data used in this study are derived from the 2004 Pelotas Birth Cohort, a long-standing, population-based cohort study. All data collection procedures were carried out by trained researchers following standardised protocols.

### Exposure: BFP at ages 6, 11 years and at both time points

During the 6-year and 11-year follow-up waves, mothers or caregivers were asked about their beneficiary status in the BFP. For the primary analysis, we identified BFP beneficiaries as families who affirmed their status as beneficiaries in both waves (N=473). In the secondary analysis, we categorised the sample into two groups: those who received benefits at one time point but not at the other. Specifically, we examined (a) individuals who were beneficiaries at age 6 but lost the benefit at age 11 (N=421) and (b) individuals who were not beneficiaries at age 6 but received the benefit at age 11 (N=172).

### Human capital outcomes at age 18

Grade repetition was assessed by the following question ‘Have you ever repeated a school grade?’, with possible answer yes/no. The category of ‘neither studying nor working’ comprises individuals who responded negatively to both questions: ‘Are you currently studying?’ and ‘Are you currently working?’, with the available response options being yes or no.

Crime occurring in the past year was evaluated through a confidential self-report questionnaire, using items developed for the Edinburgh Study of Youth Transitions and Crime.^43^ This questionnaire was previously piloted, adapted and applied in the 1993 Pelotas Birth Cohort, a concurrent longitudinal study within the same population.^44^,^46^ Three binary crime outcomes were constructed: violent crime, non-violent crime and any crime. Violent crime was measured by four items, including hitting someone on purpose with the intention of hurting or injuring them; using force, threats or violence to get money or something else from somebody; carrying a knife or other weapon for protection or to fight; and using a weapon against someone. Non-violent crime was assessed by nine items, including stealing something from a shop or store; damaging or destroying property; breaking into a car to steal something; stealing a car or motorbike; selling illegal drugs to someone; breaking into a house or building with the intention of stealing something; selling something that belonged to others or was stolen; stealing money or property that someone was carrying without using violence and setting fire or trying to set fire to something on purpose.^46^ All items were dichotomised as ‘yes’ or ‘no’. Individuals reporting any of the specified violent or non-violent behaviours were categorised as having engaged in any crime.

Tobacco use was determined by the question ‘Do you smoke at least once a week?’ with responses ‘yes’ or ‘no.’ Binge drinking was constructed by combining two questions: (a) ‘How often do you consume alcoholic beverages?’ with responses ranging from ‘Never’ to ‘Four or more times per week,’ and (b) ‘How many doses do you drink when you consume alcoholic beverages?’ with responses ranging from ‘I have never drunk before’ to ‘10 or more doses.’ Binge drinking behaviour was defined as consuming five to six doses on a single occasion.^47^

For drug use, participants were asked if they had experienced various substances, including marijuana, cocaine (snorted or injected), sleeping or stimulant pills, heroin, ecstasy, crack cocaine, LSD, inhalants, volatile substances (glue sniffing) or others. Response options included ‘I have never used it,’ ‘I have just tried once,’ ‘I have used before, but I do not use it now,’ ‘I use it sometimes,’ ‘I use it on weekends,’ and ‘I use it every day or almost every day.’ Substance use behaviour was considered if the individual reported using one or more of the substances sometimes, on weekends or every day.

### Covariates

Perinatal interview: Maternal and child characteristics at childbirth included maternal schooling (years of formal education completed), maternal age, marital status (distinguishing between single mothers and those living with a partner), skin colour/ethnicity (categorised as white, black and mixed), the number of live children and child sex (female and male).

A 4-year follow-up interview: Household wealth scores were determined by assessing household characteristics using the National Wealth Index questionnaire.^48^ Principal component analysis was applied to integrate various factors, including the household head’s education, the number of bedrooms and bathrooms (equipped with a shower and toilet), the count of televisions, vehicles and ownership (yes/no) of assets such as refrigerator, DVD or video tape, freezer/duplex refrigerator, washing machine, microwave, telephone line, computer and air conditioner. The resulting wealth index provided a comprehensive measure of a household’s economic status, facilitating the classification of households into five wealth strata based on reference cut-off values for each municipality.^49^ Employing Pelotas-specific reference values, the wealth indexes were stratified as follows: 20–280=first strata (poorest households), 281–367=second strata, 368–475=third strata, 476–618=fourth strata and 619–1478=fifth strata (wealthiest households, excluded from the analysis). While the BFP eligibility traditionally relies on per capita household income, it has been proposed that the first strata of the wealth index serve as a valuable proxy for BFP eligibility. This stratum represents the poorest population, potentially qualifying for the programme in the municipality and is considered less susceptible to temporal variability and information errors than household income.^38^

A 6-year follow-up interview: Maternal depressive symptoms were assessed using the Edinburgh Postnatal Depression Scale (EPDS).^50^ The EPDS comprises 10 items, with total scores ranging from 0 to 30. Higher scores correlate with more pronounced depressive symptoms. The presence of maternal depressive symptoms was established using a validated cut-off of ≥13, specifically endorsed for screening major depressive episodes among adults in the general population in Brazil.^51^

### Statistical analyses

To evaluate the impact of the BFP on youth human capital, we employed propensity score matching (PSM) using Stata V.14.^52 53^ PSM aids in mitigating bias related to differences in the distributions of observed covariates between BFP beneficiaries (treatment group) and non-beneficiaries (comparison group). Matching is conducted based on the subjects’ similarity in estimated treatment probabilities, known as propensity scores (PS).^54 55^ The PS in this study represents the probability of being a BFP beneficiary given the observed covariates for the child, mother and household. BFP participation covariates were selected using probit regression models, and covariates for our outcomes of interest were estimated using probit regression models. The PS was calculated using a broad set of variables that could influence BFP participation or outcomes, aiming to account for potential unobserved confounders. These included household wealth index, maternal characteristics (age at childbirth, parity, maternal schooling, skin colour and maternal depressive symptoms), and child sex.^54^

To minimise bias, we implemented 1:1 nearest-neighbour matching (NNM), pairing each beneficiary with the non-beneficiary possessing the closest PS.^54^ This matching was performed with replacement and a calliper (setting the maximum allowable distance for potential matches) of 0.005.^54 55^ NNM is widely recognised as a robust method for improving comparability between groups and reducing confounding in observational studies.^56^ Following each model, covariate balance was assessed by comparing means between the treated and comparison groups and calculating the percentage reduction in bias. Balance was considered acceptable when the bias was substantially reduced and mean differences were no longer statistically significant.^54^,^56^ Additionally, density-distribution plots of PSs for both BFP and non-BFP participants were presented. These plots allowed a visual assessment of the overlap or common support condition, ensuring that respondents with identical PS values have a positive probability of being both BFP participants and non-participants. The PSM enables the estimation of the average treatment effect on the treated (ATT). In this context, the treatment effect is calculated by averaging the difference between the outcomes of BFP beneficiaries and eligible non-beneficiaries.^54 56^ For all binary outcomes, ATT represents the absolute difference in proportions of the outcome between treated and matched control groups. We estimated 95% CIs for the ATT using bootstrapping with 1000 replications, resampling the matched sample to account for estimation uncertainty. Additionally, we adopted the language of evidence to interpret the results of our statistical tests. In this framework, p values were categorised based on their approximate range: 0.1–1.0 indicates little to no evidence, 0.05–0.1 suggests weak evidence, 0.01–0.05 represents moderate evidence, 0.001–0.01 indicates strong evidence and <0.001 reflects very strong evidence.^57^ Furthermore, separate PSs were estimated for each exposure group (BFP at age 6 only and BFP at age 11 only) to ensure appropriate matching with comparable non-beneficiaries, using the same set of covariates as in the main analysis. Detailed model specifications for the PS estimation and ATT calculation are provided in the Online supplemental material.

## Results

### Sample characteristics

Table 1 displays the sample characteristics for the current analysis, encompassing a total of 2743 individuals. Among them, 473 (26.03%) were beneficiaries of the BFP at both 6 and 11 years old, while 1344 (73.97%) individuals had never been beneficiaries at either age. A higher percentage of beneficiaries reported repeating a school grade, being neither employed nor studying, and engaging in tobacco use, drug use and non-violent crime. In contrast, a lower percentage of BFP beneficiaries reported binge drinking behaviour (table 1). Table 2 presents the covariates associated with BFP participation at ages 6 and 11. Compared with individuals who were never beneficiaries, those who received benefits at both time points were more likely to belong to the poorest wealth quintile. They were also more likely to have mothers of black or mixed ethnicity, lower maternal education levels, a higher number of live births and a greater prevalence of depressive symptoms. Child sex, maternal marital status and maternal age were not associated with BFP participation. Nonetheless, these three variables were associated with most of our outcomes of interest and therefore were included in the PSM models (online supplemental tables S1 and S2).

**Table 1:** Distribution of socioeconomic characteristics and outcomes variables among Bolsa Família Programme (BFP) participants and non-participants, 2004 Pelotas Birth Cohort (N=2743)

	Total sample	BFP at ages 6 and 11 years	Non-BFP at age 6 and 11 years
	N or mean (% or SD)	N or mean (% or SD)	N or mean (% or SD)
Female child	1364 (49.69)	250 (52.85)	653 (48.59)
Wealth score			
Wealth index (quintile)	351.05 (116.90)	293.16 (106.88)	388.44 (109.97)
First quintile (poorest)	596 (21.71)	183 (38.69)	162 (12.05)
Second to fourth quintile	2149 (78.29)	290 (61.31)	1182 (87.95)
Maternal schooling (years)	7.50 (3.07)	5.73 (2.55)	8.56 (2.90)
Maternal skin colour			
White	1897 (69.11)	280 (59.20)	1006 (74.85)
Black/mixed	848 (30.89)	193 (40.80)	338 (25.15)
Mother not living with partner at childbirth	473 (17.23)	81 (17.12)	230 (17.11)
Maternal parity at childbirth	1.39 (1.68)	2.22 (2.04)	0.94 (1.25)
Mother’s age at childbirth (years)	25.47 (6.72)	26.01 (6.77)	25.43 (6.71)
Maternal depressive symptoms at age 6	420 (18.30)	110 (25.11)	153 (12.68)
Youth outcomes at age 18 years:			
Have repeated a school grade	1616 (59.09)	355 (75.21)	641 (47.87)
Neither working or studying	178 (7.06)	34 (8.02)	67 (5.36)
Non-violent crime	137 (4.62)	25 (6.31)	48 (4.10)
Violent crime	419 (13.39)	52 (12.35)	170 (13.80)
Any crime	474 (16.08)	60 (15.42)	189 (16.28)
Tobacco use	411 (12.61)	67 (14.76)	153 (11.95)
Binge drinking	493 (21.55)	66 (20.82)	215 (24.00)
Drug use	431 (16.00)	55 (15.99)	157 (14.46)
Total	2743 (100.00)	473 (26.03)	1344 (73.97)

N, number of observations.

**Table 2:** Association between baseline covariates and Bolsa Família Programme at ages 6 and 11 (N=1817)

	Coefficient (SE)	95% CI	P value
At ages 6 and 11 years			
Female child	0.10 (0.05)	−0.02 to 0.22	0.110
First wealth quintile (poorest)	0.93 (0.07)	0.77 to 1.08	<0.001
Maternal schooling	−0.21 (0.01)	−0.23 to −0.19	<0.001
Black/mixed mothers	0.43 (0.06)	0.29 to 0.56	<0.001
Mother not living with partner at childbirth	0.00 (0.08)	−0.16 to 0.16	0.310
Maternal parity at childbirth	0.28 (0.02)	0.25 to 0.32	<0.001
Maternal age at childbirth	−0.01 (0.00)	−0.01 to 0.00	0.104
Maternal depressive symptoms	0.51 (0.07)	0.33 to 0.67	<0.001

### PSM balancing results

**Figure 1:**
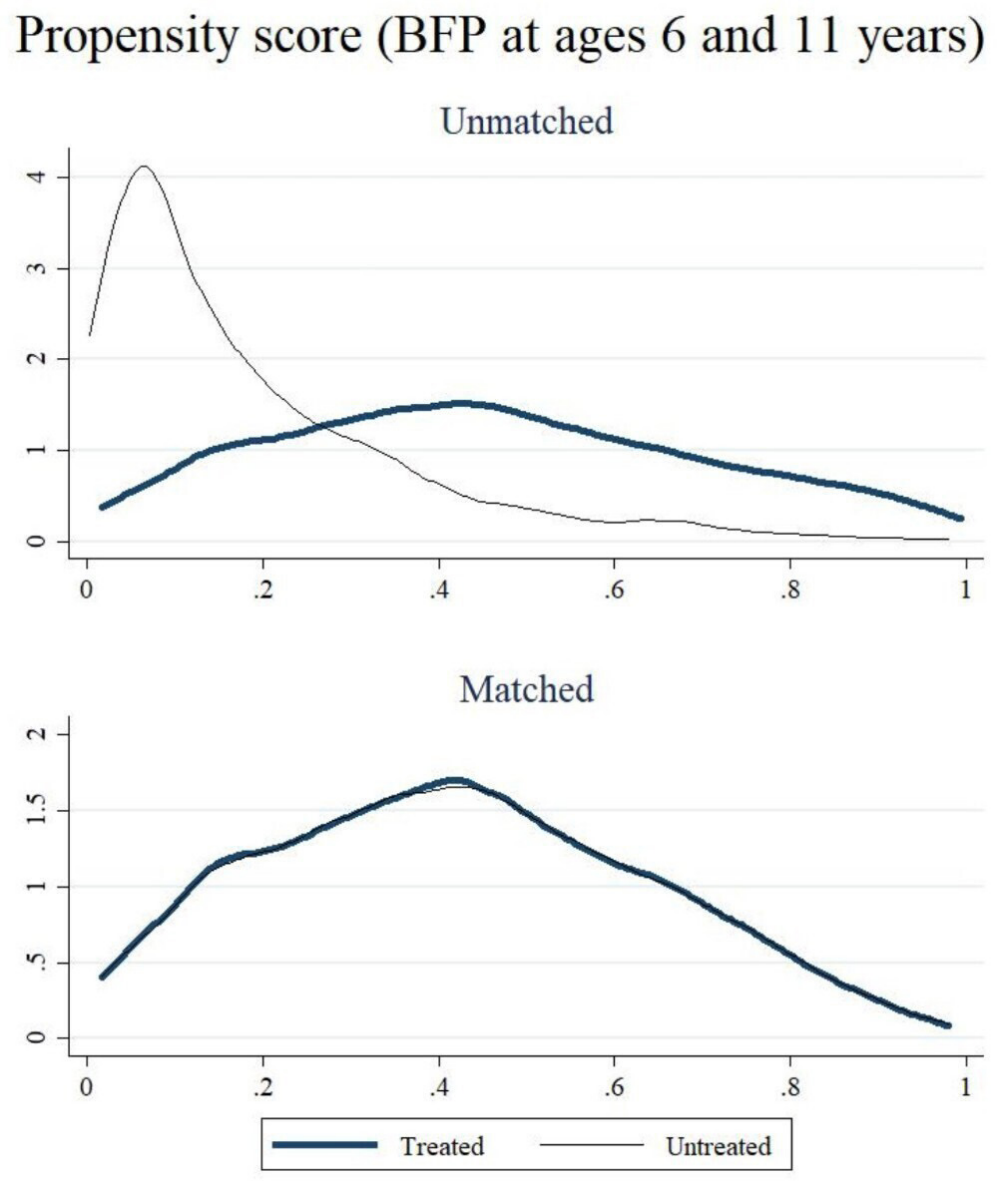
Distribution of the propensity score before and after matching (overlap assumption), matching for sex, maternal skin colour, maternal schooling, household wealth score, maternal age at childbirth, maternal depressive symptoms at age 6 years and parity. Bolsa Família Programme (BFP) at age 6 and 11 years.

Figure 1 and online supplemental table S3 show the balance of covariates before and after implementing PSM using the NNM technique. The mean differences between groups were consistently non-significant, indicating the successful elimination of disparities in the distributions of covariates. The overlap of the PS between matched beneficiaries and non-beneficiaries, as shown in figure 1, indicates that each matched pair had an equal probability of being eligible for BFP based on observed covariates. The same pattern was found for the secondary analysis (ie, BFP at age 6 only and at age 11 only, (online supplemental figures S2 and S3). Online supplemental table S4 details the number of cases included (on support) and excluded (off support) in the PSM process, along with the percentage of bias reduced after PSM. The bias reduction ranged between 79.9% and 89.0%.

### Effects of BFP on youth human capital

Table 3 presents the ATT of BFP participation at ages 6 and 11 on youth human capital outcomes at age 18. We found no evidence that BFP influenced the likelihood of being neither studying nor working (ATT 0.01, 95% CI −0.04 to 0.06, p=0.638), tobacco use (ATT −0.03, 95% CI −0.10 to 0.05, p=0.406), binge drinking (ATT −0.05, 95% CI −0.15 to 0.05, p=0.293), violent crime (ATT 0.00, 95% CI −0.08 to 0.07, p=0.936) or any crime (ATT −0.06, 95% CI −0.14 to 0.03, p=0.128).

**Table 3:** Effects of BFP (at ages 6 and 11 years) on 18-year-old youth outcomes* (N=1817)

	BFP vs non-BFP difference		
	ATT (95% CI)	Bootstrap SE	P value
BFP at ages 6 and 11 years			
Have repeated a school grade	**0.07 (−0.02 to 0.16**)	**0.04**	**0.097**
Neither studying nor working	0.01 (−0.04 to 0.06)	0.02	0.638
Non-violent crime	**−0.04 (−0.09 to 0.01**)	**0.02**	**0.072**
Violent crime	−0.00 (−0.08 to 0.07)	0.03	0.936
Any crime	−0.06 (−0.14 to 0.03)	0.04	0.128
Tobacco use	−0.03 (−0.10 to 0.05)	0.03	0.406
Binge drinking	−0.05 (−0.15 to 0.05)	0.05	0.293
Drug use	**−0.07 (−0.17 to 0.02**)	**0.04**	**0.075**

Only p-values indicating at least weak evidence are in bold (ie, p<0.10). Evidence-strength guide: 0.10–1.00 = little to no evidence (not bold); 0.05–0.10 = weak; 0.01–0.05 = moderate; 0.001–0.01 = strong; <0.001 = very strong.

* Matching for sex, maternal skin colour, maternal schooling, household wealth score, maternal age at childbirth, maternal depressive symptoms at age 6 years and number of live births.

ATT, Average Treatment Effect on the Treated; BFP, Bolsa Família Programme.

However, we found weak evidence suggesting that BFP may be associated with a higher likelihood of school grade repetition (ATT 0.07, 95% CI −0.02 to 0.16, p=0.097) and lower likelihood of engaging in non-violent crime (ATT −0.04, 95% CI −0.09 to 0.01, p=0.072) and drug use (ATT −0.07, 95% CI −0.17 to 0.02, p=0.075). In other words, on average, BFP participation was associated with a slightly higher likelihood of repeating a school grade but a lower likelihood of engaging in non-violent crime and reporting drug use.

Online supplemental table S5 presents the effect of BFP at a single time point (ie, either at age 6 years but not at age 11 years, or at age 11 years but not at age 6 years) on youth outcomes at age 18. Separate PSs were estimated for each exposure group to match treated individuals with comparable non-beneficiaries. The analysis did not provide any evidence to support the influence of BFP on any measured outcome. This suggests that the effect of BFP on youth outcomes may be dependent on the duration of BFP participation.

## Discussion

Using a longitudinal design and PSM, we examined the impact of BFP participation at ages 6 and 11 on youth human capital outcomes at age 18 in the 2004 Pelotas Birth Cohort. We found no evidence that BFP influenced educational or employment status, tobacco use, binge drinking or violent crime. However, our findings suggest that BFP recipients may be more likely to have repeated a school grade but less likely to engage in non-violent crime or report drug use.

The finding that BFP is associated with a higher likelihood of grade repetition contrasts with previous studies that highlight its positive effects on educational outcomes, including increased school enrolment, higher attendance and improved progression rates.^958^,^62^ On the other hand, other studies reported no evidence of effects of BFP on educational outcomes.^5 63^ The mixed findings demonstrate that the existing literature on whether BFP has impacted educational outcomes, particularly grade progression, requires further investigation, as the evidence varies depending on the chosen time period, dataset and evaluation method. An initial hypothesis could be that students who would have dropped out of school early, but remain enrolled due to the BFP conditionality, may be more likely to repeat a grade. The findings of Simões and Sabates align with this hypothesis, indicating that while the BFP helps reduce grade repetition and school dropout rates, students benefiting from the programme tend to underperform in core subjects such as mathematics and Portuguese.^62^ Two systematic reviews evaluated the effects and impact of the BFP on educational indicators. The first review included 12 studies published between 2004 and 2014.^61^ The findings showed that while BFP had no discernible impact on academic proficiency and performance, it significantly influenced school attendance and dropout rates. Moreover, there was evidence that BFP had positive outcomes on educational indicators particularly for females and for the Northern region of Brazil.^61^ The more recent review also encompassed 12 studies, but these were published from 2010 to 2020.^9^ The findings based on quantitative studies showed that while BFP had no discernible impact on academic proficiency and performance, it significantly influenced school attendance and dropout rates. Moreover, there was evidence that BFP had positive outcomes on educational indicators particularly for females and for the Northern region of Brazil (50) and qualitative studies based on beneficiaries’ perspectives suggested that they perceived the BFP as contributing to the academic performance of children and adolescents in school (9). These findings underscore that the impact of BFP may be more pronounced in subgroups of the population that could benefit the most from CCTs, such as girls and individuals from more economically disadvantaged regions of Brazil.^4^ Additionally, Paiva *et al*^5^ showed that BFP coverage was not associated with school progression and drop-out rates at the municipal level. However, school attendance monitoring (ie, conditionalities monitoring) was negatively associated with dropout rates and positively associated with school progression. The authors discuss that the cash transfer may represent the only stable source of income for the family, and its contingency on school attendance makes conditionality monitoring an important predictor of schooling outcomes. Based on this, we hypothesise that the BFP may improve school enrolment and attendance rates (due to the conditionality), which are not directly linked to learning outcomes associated with grade progression. Moreover, improvements in school quality and school environment are not addressed by CCTs like BFP. Public policy interventions should not solely focus on combating grade repetition per se, as merely increasing a student’s years spent in the classroom may not be sufficient to improve academic performance.^61^ Therefore, an alternative hypothesis is that there are subjective ways of interpreting academic performance that go beyond grades and grade repetition, which are the primary measures used in quantitative studies.

For crime-related outcomes, we found evidence that BPF is associated with a lower probability of committing non-violent crimes. This result is in line with a previous study showing that BFP coverage is associated with reduced crime, particularly economically motivated crimes such as robbery.^64^ Similarly, other studies suggested that BFP expenditures contribute to reduction in robbery, theft, kidnapping and murder rates at the municipal level in Brazil.^65 66^ Chioda *et al*^64^ proposed that potential mechanisms linking CCT programmes and lower crime rates include increased household income, reorganised family routines and changes in peer group and social networks.^64^ In addition, Breckin,^67^ based on the social control theory within a criminology framework, discusses that the crime prevention effect of CCTs may result from strengthened social bonds (with educational and other institutions), leading to a lower propensity for committing crime. Considering the close connection between education, childhood exposure to socioeconomic disadvantages and later criminal behaviour,^26 44 45 68^ examining the effectiveness of CCTs in reducing adolescent involvement in crime is crucial for public policy evaluation. Despite the significant relevance of studying the effects of BFP in preventing crime, few studies exist on this topic in Brazil,^64^,^66^ particularly using individual-level data. Our study addresses this gap in the literature and highlights the need for further investigation into the effect of CCTs on crime rates in low- and middle-income countries (LMICs), with a particular focus on the mechanisms underlying this association.^67^

Our findings suggest that, compared with eligible but never beneficiaries, the BFP beneficiaries at ages 6 and 11 are less likely to report drug use. Drug use during adolescence and young adulthood is associated with long-term consequences for neurobiological functions, leading to cognitive and behavioural impairments.^69 70^ Additionally, exposure to drugs during this developmental period increases the likelihood of developing a future substance use disorder.^71^ Previous evidence showed that episodic and frequent patterns of substance use are strongly associated with lower childhood socioeconomic status and lower parental education level.^72^,^74^ However, as far as we are aware, only one study examined the impacts of CCTs on drug use among beneficiaries. The ‘Family Rewards’ programme, a CCT implemented in New York City (USA), offered cash assistance to low-income families to reduce economic hardship in three key areas: children’s education, family preventive healthcare and parents’ employment.^75^ Findings based on the randomised assignment method of the study revealed that Family Rewards led to significant decreases in adolescent substance use rates among programme group adolescents and their peers, compared with adolescents in the control condition.^75^ We have not found any studies using samples from LMICs or BFP beneficiaries. Nonetheless, Morris *et al* and our findings suggest that the potential effects of CCTs in preventing substance use during individuals’ formative years may represent a promising strategy for mitigating the societal consequences of substance abuse.

This study enhances current understanding of the long-term impacts of CCTs during childhood on early adulthood outcomes through the examination of a population-based birth cohort data from a middle-income country. To the best of our knowledge, this is the first study to examine the longitudinal effects of receiving BFP, spanning childhood and preadolescence, on a broad spectrum of human capital outcomes including education, employment, drug and alcohol use, and involvement in crime. Furthermore, many prior studies have relied on data from the programme’s first decade of implementation, yielding results primarily focused on early childhood, using cross-sectional or municipal-level analyses. In contrast, our study used individual, updated longitudinal data with outcomes assessed in early adulthood. Additionally, we applied PSM to mitigate potential selection bias and confounding variables. Nonetheless, it is important to interpret the findings cautiously, as several limitations warrant consideration. First, the interpretation of the treatment effect estimates derived from PSM analyses assumes that there are no unobserved confounding variables. Despite using PSM to balance covariates, any factors not captured by the model may lead to residual selection bias. Second, our measures of grade repetition and ‘neither studying nor working’ were evaluated through retrospective questions with a binary yes/no response format. Consequently, we were unable to assess factors such as timing, frequency and reasons associated with grade repetition, for instance. Additionally, we lacked detailed information for enhancing comprehension of the effects of BFP, such as the amount of benefit received and compliance with conditionalities. In particular, we did not have data on first-stage outcomes related to compliance, such as vaccination coverage, nutritional monitoring or detailed school enrolment and attendance. These indicators would provide valuable insights into the mechanisms through which the programme may (or may not) influence long-term human capital development. Future research may explore these factors among BFP beneficiaries to gain a more comprehensive understanding of CCTs in human capital accumulation. We also note that our analyses relied on self-reported programme participation, as our dataset did not include alternative eligibility proxies or repeated pretreatment outcome measures. These constraints prevented us from implementing additional robustness checks, such as using survey-based proxies of eligibility or combining PSM with difference-in-differences techniques. While beyond the scope of the present study, such strategies would be valuable to strengthen the design and should be pursued in future research. Furthermore, although we attempted to capture heterogeneity by distinguishing between benefit receipt at ages 6, 11 or both, we were not able to construct a continuous measure of treatment duration across childhood and adolescence. Such an approach would allow for a more precise assessment of exposure length, but requires more granular longitudinal data on programme receipt than was available in our dataset. Finally, Brazil is an enormous country both in size and population, with marked economic inequalities across regions. We acknowledge that the Pelotas cohort displays regional characteristics that may limit the external validity of our findings. Pelotas differs from the national average in income, human development and crime rates, as well as in the trajectory of Bolsa Família coverage.^19 38^ Therefore, our results should be interpreted as context-specific to Pelotas and not generalised across Brazil’s diverse regions and populations. As suggested by previous research, there may be regional effects of BFP,^11 61^ so replication of our findings using data derived from other Brazilian regions is needed.

The evidence of the current study highlights the relevance of outcomes not explicitly addressed by the BFP. Specifically, while the programme primarily aims to reduce family poverty and support early child health and education, it also envisions broader, long-term benefits. Our results indicate that, beyond its direct focus on outcomes such as school attendance and immunisation, the incentives provided by CCTs through the BFP can influence other important aspects of youth health behaviour, such as substance use and crime involvement, with lasting effects.

## Supplementary material

10.1136/bmjph-2025-003192 online supplemental file 1

## Data Availability

Data are available on reasonable request.
