# Supplementary material for: Brazilian conditional cash transfer programme’s impact on youth human capital outcomes: the 2004 Pelotas Birth Cohort
Source: BMJ Public Health. 2025 Nov 10;3(2):e003192. doi: 10.1136/bmjph-2025-003192 (PMC12606488; doi:10.1136/bmjph-2025-003192)
Supplement: online supplemental file 1 [file bmjph-3-2-s001.docx]

**Supplementary Material**

**Brazilian conditional cash transfer programme’s impact on youth human capital outcomes**

Jessica Mayumi Maruyama, Cristiane Silvestre de Paula, Carolina Andrea Ziebold Jorquera, Luciana Tovo-Rodrigues, Iná S. Santos, Aluísio J. D. Barros, Joseph Murray, Sara Evans-Lacko, Alicia Matijasevich

Supplementary methods. Propensity Score Matching (PSM) Model Specifications

Figure S1. Flowchart of included sample

Figure S2. Distribution of the propensity score before and after matching (overlap assumption), matching for sex, maternal skin colour, maternal schooling, household wealth score, maternal age at childbirth, maternal depressive symptoms at age 6 years, and parity. Bolsa Família Programme (BFP) at age 6 but not at age 11 years.

Figure S3. Distribution of the propensity score before and after matching (overlap assumption), matching for sex, maternal skin colour, maternal schooling, household wealth score, maternal age at childbirth, maternal depressive symptoms at age 6 years, and parity. Bolsa Família Programme (BFP) at age 11 but not at age 6

Table S1. Bivariate association between baseline characteristics and youth outcomes (have repeated a school grade, neither working nor studying, non-violent crime, violent crime, and any crime) at age 18 years

Table S2. Bivariate association between baseline characteristics and youth outcomes (tobacco use, binge drinking, and drug use) at age 18 years

Table S3. Covariates balance before and after nearest-neighbor propensity score matching (BFP at ages 6 and 11 years)

Table S4. Common support and bias reduction after nearest-neighbour propensity score matching

Table S5. Effects of BFP (at age 6 years but not at age 11 years, and at age 11 years but not at age 6 years) on 18-year-old youth outcomes

**Supplementary methods. Propensity Score Matching (PSM) Model Specifications**

**Propensity Score Estimation**
The propensity score for individual *i* was estimated using a probit regression model:

$$P\left( T_{i}=1 | x_{i} \right)= \phi(\beta_{0}+ \beta_{1} X_{1i}+\ldots+ \beta_{k}X_{ki}$$

where T_i​_ indicates Bolsa Família Program (BFP) participation, X_1i_, …X_ki_ are covariates (household wealth index, maternal age at childbirth, parity, maternal schooling, skin color, depressive symptoms, and child sex), and Φ is the cumulative normal distribution. Separate propensity scores were estimated for BFP participation at ages 6, 11, and both ages.

**Matching Algorithm**

We applied 1:1 nearest-neighbour matching with replacement, using a caliper of 0.005. Each beneficiary was paired with the non-beneficiary having the closest propensity score within the caliper.

**Average Treatment Effect on the Treated (ATT)**

The ATT was estimated as:

$$ATT=E\left[ Y\left( 1 \right)-Y\left( 0 \right) | T=1 \right]$$

where Y(1) is the outcome if treated, and Y(0) is the counterfactual outcome estimated through matched controls. For binary outcomes, the ATT corresponds to the absolute difference in proportions between treated and matched control groups.

**Statistical Inference**

We calculated 95% confidence intervals using bootstrapping with 1,000 replications, resampling the matched sample to account for uncertainty.

| 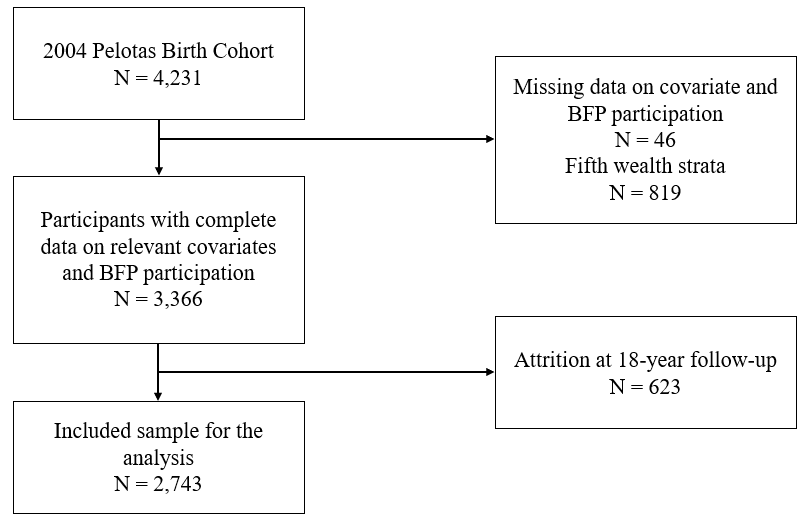 |
| --- |
| Figure S1. Flowchart of included sample, 2004 Pelotas Birth Cohort |

|  |
| --- |
| Figure S2. Distribution of the propensity score before and after matching (overlap assumption), matching for sex, maternal skin colour, maternal schooling, household wealth score, maternal age at childbirth, maternal depressive symptoms at age 6 years, and parity. *Bolsa Família* Programme (BFP) at age 6 but not at age 11 years. |

|  |
| --- |
| Figure S3. Distribution of the propensity score before and after matching (overlap assumption), matching for sex, maternal skin colour, maternal schooling, household wealth score, maternal age at childbirth, maternal depressive symptoms at age 6 years, and parity. *Bolsa Família* Programme (BFP) at age 11 but not at age 6 |

| Table S1. Bivariate association between baseline characteristics and youth outcomes (have repeated a school grade, neither working nor studying, non-violent crime, violent crime, and any crime) at age 18 years (N = 2,743) | | | | | | | | | | |
| --- | --- | --- | --- | --- | --- | --- | --- | --- | --- | --- |
|  | Have repeated a school grade | p | Neither working nor studying | p | Non-violent crime | p | Violent crime | p | Any crime | p |
|  | OR (95% CI) |  | OR (95% CI) |  | OR (95% CI) |  | OR (95% CI) |  | OR (95% CI) |  |
| Female child | 0.65 (0.57;0.75) | <0.001 | 0.92 (0.69;1.22) | 0.558 | 0.54 (0.36; 0.80) | 0.003 | 0.68 (0.53; 0.84) | 0.001 | 0.68 (0.54; 0.84) | 0.001 |
| Wealth index (2nd to 5th quintile: reference) |  |  |  |  |  |  |  |  |  |  |
| 1st quintile (poorest) | 3.51 (2.86;4.29) | <0.001 | 2.29 (1.67;3.15) | <0.001 | 1.22 (0.77; 1.92) | 0.387 | 1.20 (0.91; 1.56) | 0.188 | 1.24 (0.95; 1.60) | 0.102 |
| Maternal schooling (years) | 0.78 (0.76;0.79) | <0.001 | 0.87 (0.83;0.91) | <0.001 | 0.93 (0.87; 0.99) | 0.029 | 0.95 (0.91; 0.99) | 0.013 | 0.94 (0.91; 0.98) | 0.004 |
| Maternal skin color (White: reference) |  |  |  |  |  |  |  |  |  |  |
| Black/mixed | 2.17 (1.85;2.53) | <0.001 | 1.42 (1.05;1.93) | 0.022 | 1.32 (0.88; 1.97) | 0.184 | 1.24 (0.98; 1.58) | 0.070 | 1.32 (1.05; 1.67) | 0.016 |
| Mother not living with a partner at childbirth | 1.52 (1.26;1.83) | <0.001 | 1.28 (0.89;1.85) | 0.175 | 1.64 (1.04; 2.60) | 0.033 | 1.58 (1.19; 2.08) | 0.001 | 1.64 (1.29; 2.14) | 0.001 |
| Maternal parity at childbirth | 1.29 (1.23;1.35) | <0.001 | 1.19 (1.11;1.28) | <0.001 | 1.11 (1.00; 1.23) | 0.045 | 1.01 (0.94; 1.08) | 0.687 | 1.02 (0.96; 1.09) | 0.460 |
| Mother`s age at childbirth (years) | 0.96 (0.95;0.97) | <0.001 | 0.99 (0.96;1.01) | 0.366 | 1.00 (0.97; 1.03) | 0.961 | 0.98 (0.97; 1.00) | 0.230 | 0.99 (0.97; 1.01) | 0.243 |
| Maternal depressive symptoms at age 6 | 2.10 (1.70;2.59) | <0.001 | 1.14 (0.75;1.74) | 0.547 | 1.57 (0.96; 2.60) | 0.073 | 1.48 (1.10; 2.00) | 0.009 | 1.43 (1.07; 1.91) | 0.016 |
| Note. 95% CI – 95% Confidence Interval | | | | | | | | | | |

| Table S2. Bivariate association between baseline characteristics and youth outcomes (tobacco use, binge drinking, and drug use) at age 18 years (N = 2,743) | | | | | | |
| --- | --- | --- | --- | --- | --- | --- |
|  | Tobacco use | p | Binge drinking | p | Drug use | p |
|  | OR (95% CI) |  | OR (95% CI) |  | OR (95% CI) |  |
| Female child | 1.07 (0.86; 1.33) | 0.525 | 0.62 (0.50; 0.76) | <0.001 | 0.94 (0.75; 1.16) | 0.574 |
| Wealth index (2nd to 5th quintile: reference) |  |  |  |  |  |  |
| 1st quintile (poorest) | 1.73 (1.34; 2.23) | <0.001 | 1.22 (0.93; 1.59) | 0.141 | 1.20 (0.90; 1.59) | 0.202 |
| Maternal schooling (years) | 0.90 (0.87; 0.93) | <0.001 | 1.01 (0.98; 1.05) | 0.357 | 1.01 (0.97; 1.04) | 0.546 |
| Maternal skin color (White: reference) |  |  |  |  |  |  |
| Black/mixed | 1.03 (0.81; 1.31) | 0.802 | 1.17 (0.93; 1.47) | 0.165 | 1.09 (0.855; 1.39) | 0.480 |
| Mother not living with a partner at childbirth | 1.30 (0.98; 1.72) | 0.060 | 1.38 (1.06; 1.80) | 0.016 | 1.52 (1.16; 2.01) | 0.002 |
| Maternal parity at childbirth | 1.10 (1.03; 1.17) | 0.001 | 0.97 (0.91; 1.04) | 0.424 | 1.06 (0.99; 1.13) | 0.080 |
| Mother`s age at childbirth (years) | 0.97 (0.96; 0.99) | 0.002 | 0.98 (0.97; 0.99) | 0.039 | 0.98 (0.97; 1.00) | 0.181) |
| Maternal depressive symptoms at age 6 | 1.38 (1.03; 1.84) | 0.027 | 0.99 (0.74; 1.31) | 0.949 | 1.13 (0.83; 1.53) | 0.432 |
| Note. 95% CI – 95% Confidence Interval | | | | | | |

| Table S3. Covariates balance before and after nearest-neighbor propensity score matching (BFP at ages 6 and 11 years) (N = 2,743) | | | | | |
| --- | --- | --- | --- | --- | --- |
|  |  | BFP, mean | Non-BFP, mean | Bias reduction (%) | T-test, p-value |
| Female child | Before | 0.51 | 0.48 |  |  |
|  | After | 0.50 | 0.53 | 17.8 | -0.75, p = 0.454 |
| 1st wealth quintile (poorest) | Before | 0.32 | 0.21 |  |  |
|  | After | 0.32 | 0.35 | 74.3 | -0.79, p = 0.430 |
| Maternal schooling | Before | 5.82 | 8.62 |  |  |
|  | After | 6.08 | 5.97 | 96.3 | 0.55, p = 0.582 |
| Black/mixed mothers | Before | 0.40 | 0.25 |  |  |
|  | After | 0.39 | 0.45 | 65.2 | -1.44, p = 0.150 |
| Mother not living with partner at childbirth | Before | 0.18 | 0.17 |  |  |
|  | After | 0.18 | 0.17 | 0.00 | 0.30, p = 0.768 |
| Maternal parity at childbirth | Before | 2.15 | 0.89 |  |  |
|  | After | 1.85 | 1.72 | 88.8 | 1.15, p = 0.252 |
| Maternal age at childbirth | Before | 25.85 | 25.36 |  |  |
|  | After | 25.43 | 24.87 | -12.4 | 1.08, p = 0.280 |
| Maternal depressive symptoms | Before | 0.25 | 0.12 |  |  |
|  | After | 0.22 | 0.22 | 99.0 | 0.07, p = 946 |
| Note. BFP – *Bolsa Família* Programme | | | | | |

| Table S4. Common support and bias reduction after nearest-neighbour propensity score matching^*^ (N = 1,817) | | | | | | | |
| --- | --- | --- | --- | --- | --- | --- | --- |
|  | Matched BFP | | Matched non-BFP | | Bias | | Bias reduction  (%) |
|  | On support | Off support | On support | Off support | Before matching | After matching |  |
|  | N | N | N | N | Mean | Mean |  |
| **BFP at ages 6 and 11 years** |  |  |  |  |  |  |  |
| Have repeated a school grade | 356 | 36 | 1126 | 0 | 37.7 | 5.5 | 85.4 |
| Neither working or studying | 388 | 50 | 1202 | 0 | 37.9 | 7.6 | 79.9 |
| Non-violent crime | 325 | 43 | 1058 | 0 | 38.2 | 4.9 | 87.2 |
| Violent crime | 361 | 32 | 1110 | 0 | 38.0 | 5.1 | 86.6 |
| Any crime | 324 | 38 | 1048 | 0 | 37.9 | 5.8 | 84.7 |
| Tobacco use | 387 | 33 | 1151 | 0 | 38.2 | 4.4 | 88.5 |
| Binge drinking | 264 | 33 | 798 | 0 | 38.6 | 4.5 | 88.3 |
| Drug use | 264 | 50 | 974 | 0 | 39.3 | 4.3 | 89.0 |
| Note. BFP – Bolsa Família Programme, N – number of participants  ^*^ Matching for sex, maternal skin colour, maternal schooling, household wealth score, maternal age at childbirth, maternal depressive symptoms at age 6 years, and parity | | | | | | | |

| Table S5. Effects of BFP (at age 6 years but not at age 11 years, and at age 11 years but not at age 6 years) on 18-year-old youth outcomes^*^ | | | |
| --- | --- | --- | --- |
|  | **BFP vs non-BFP difference** | | |
|  | ATT (95% CI) | Bootstrap SE | p |
| **BFP at age 6 years but not at age 11 years (N = 421)** |  |  |  |
| Have repeated a school grade | 0.03 (-0.06; 0.11) | 0.04 | 0.490 |
| Neither studying nor working | 0.02 (-0.02; 0.07) | 0.02 | 0.226 |
| Non-violent crime | 0.01 (-0;03; 0.05) | 0.02 | 0.568 |
| Violent crime | 0.01 (-0.05; 0.08) | 0.03 | 0.624 |
| Any crime | 0.01 (-0.06; 0.08) | 0.03 | 0.779 |
| Tobacco use | 0.01 (-0.05; 0.07) | 0.03 | 0.779 |
| Binge drinking | -0.06 (-0.15; 0.04) | 0.04 | 0.165 |
| Drug use | -0.04 (-0.12; 0.04) | 0.03 | 0.267 |
| **BFP at age 11 years but not at age 6 years (N = 172)** |  |  |  |
| Have repeated a school grade | 0.04 (-0.09; 0.17) | 0.05 | 0.484 |
| Neither studying nor working | 0.03 (-0.05; 0.11) | 0.03 | 0.379 |
| Non-violent crime | 0.00 (-0.06; 0.06) | 0.02 | 0.999 |
| Violent crime | 0.07 (-0.04;0.18) | 0.04 | 0.131 |
| Any crime | 0.01 (-0.11; 0.13) | 0.05 | 0.881 |
| Tobacco use | -0.02 (-0.12; 0.08) | 0.05 | 0.652 |
| Binge drinking | 0.02 (-0.13; 0.17) | 0.06 | 0.741 |
| Drug use | 0.02 (-0.10; 0.13) | 0.05 | 0.718 |
| Note. BFP – *Bolsa Família* Programme; 95% CI – 95% Confidence Interval; SE – Standard Error  ^*^ Matching for sex, maternal skin colour, maternal schooling, household weath score, maternal age at childbirth, maternal depressive symptoms at age 6 years, and parity | | | |
